# Functionalization of clay surface for the removal of uranium from water

**DOI:** 10.1016/j.mex.2021.101275

**Published:** 2021-02-19

**Authors:** Li Bao, Fuyu Guo, Hanrui Wang, Steven L. Larson, John H. Ballard, Heather M. Knotek-Smith, Qinku Zhang, Jing Nie, Ahmet Celik, Saiful M. Islam, Shalom Dasari, Naiming Zhang, Fengxiang Han

**Affiliations:** a Department of Chemistry, Physics and Atmospheric Science, Jackson State University, Jackson, MS 39217, USA; b Yunnan Agricultural University, Kunming 650201, China; c U.S. Army Engineer Research and Development Center, Vicksburg, MS 39180-6199, USA

**Keywords:** Clay minerals, Phosphoric acid, Surface functionalization, Adsorption, Uranium removal

## Abstract

A modification method of clay mineral surface was developed to improve its adsorption capacity of uranium. Uranium is a radionuclide with high toxicity and extremely long half-life, which can pollute the environment and endanger human health. This study proposes a new method of activation of clay mineral surface with phosphoric acid for rapid adsorption of uranium from aqueous solution. Compared with other modification methods, this method has the advantages of availability of raw materials, simple operation and good adsorption effects. It provides a cost-effective material to capture uranium ions from water. The essences of this new development are as following:

• Activation and changes of clay minerals’ surface functionalities with the treatment of phosphoric acid

• Controlled modifications of the surface properties of the clay towards the enhancement of U adsorption capacity

• Rapid removal of uranium from water

## Graphical abstract

**Figure:**
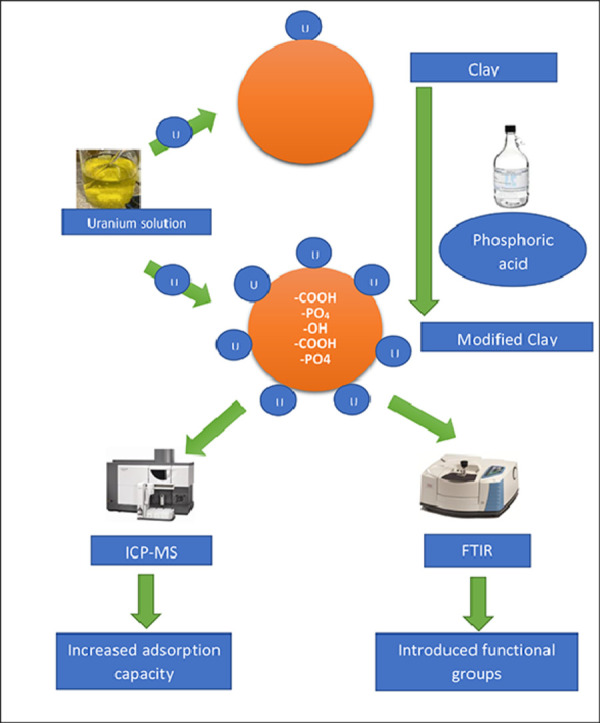

Specifications table

**Table:** 

Subject Area	*Environmental Science*
More specific subject area	*Heavy metal pollution control and remediation*
Method name	*Functionalization of Clay Surface for the Removal of Uranium from Water*
Name and reference of original method	*Grabias, E., Gładysz-Płaska, A., Książek, A., & Majdan, M. (2014). Efficient uranium immobilization on red clay with phosphates. Environmental chemistry letters, 12(2), 297-301.*
Resource availability	*N/A*

## Method details

### Background

This research found that direct phosphoric acid-activated clay can greatly increase the adsorption capacity of clay for uranium. This is a simple, low-cost clay modification method that has not been found in other researches. Uranium-containing wastewater is generated in mining and industrial areas, nuclear energy and weapon manufacturing facilities, and battlefields [1]. Uranium is a radioactive element that exhibits several isotopes. Among them U^235^ and U^238^ attain a natural abundance of 0.72% and 99.27% and possess a half-life, 7.04 × 10^8^ y and 4.468 × 10^9^ y, respectively [2]. The radioisotopes are extremely toxic and can cause cumulative damage to the biological systems including human body. The exposure to organisms to a large doses of radiation causes various diseases and sometimes even lead to death [3]. Even if surviving, the radiation also causes genetic mutations with irreversible harm. In addition, U is highly chemically toxic. After entering the human body, uranium accumulates in the bones, liver and kidneys and long-term accumulation causes liver and kidney function lesions [4]. The long half-lives, as well as their radioactive decay to radium and radon and their gamma radioactivity exacerbates the harm of uranium-containing wastewater to the environment [5].

Clay minerals are cost-effective materials that can be used to solve the problem of uranium pollution [6]. However, the adsorption capacity of most clay minerals is not significant. Therefore, it is necessary to modify clay minerals via functioning surfaces to improve the adsorption efficiency of uranium. Phosphate as a modifier has the advantages of environmental friendliness and low cost compared with the other organic modifiers and the affinity of phosphate for uranium is good [7].

Phosphate has a high binding affinity for uranium. Use phosphoric acid to modify clay minerals and introduce phosphoric group to activate the surface of the clay minerals and increase acidic functional groups with a low cost. According to the Lewis acid-base theory, uranium tends to chelate and coordinate with oxygen-containing anions to form stable coordination bonds [8]. Hence, oxygen atoms in the phosphate group chelate with uranium [9]. However, phosphate itself is easily soluble in water. Even if phosphate is coordinated with uranium in the solution, it is difficult to separate U from the solution [10]. Therefore, we activated the surfaces of clay minerals with phosphate first. The phosphate group was anchored on the surface and between the layers of clay minerals. Then the activated clay minerals with phosphoric acid were used to adsorb uranium in the aqueous solution. This method made it easier to separate the uranium from aqueous solution after adsorption [11]. In addition, phosphoric acid is a low-cost common raw material. Moreover, phosphoric acid is more environmentally friendly than most organic modifiers as a plant nutrient in soil and water.

In this study, phosphoric acid-activated clay minerals were synthesized and its efficiencies on the adsorption of uranium from aqueous solution was studied.

### Procedures

1. Weigh about 0.10 g of clay minerals in a beaker.
2. Add 1.0 mol/L phosphoric acid solution to the beaker.
3. Place the beaker on a shaker and shake sample at 100 rpm at room temperature for one hour.
4. Equilibrate the samples at room temperature for one hour.
5. Solution was separated from the phosphoric acid adsorbed clay
6. Add 200 mg/L uranium solution to the sample.
7. Place the beaker on a shaker and shake sample at 100 rpm at room temperature for one hour.
8. Equilibrate the sample at room temperature for one hour, centrifuge the mixture and separate the supernatants.
9. Take the supernatant and determine the uranium concentration with ICP-MS.
10. The sample was dried in an oven at 110 °C and the FTIR (Fourier Transform Infrared Spectroscopy) of the sample was measured.

### Final remarks

The uranium adsorption capacity of phosphoric acid activated clay and original clay is significantly different at *p* = 0.05. This indicates that adding phosphoric acid significantly improved the uranium adsorption on clay minerals. Phosphoric acid activated clay minerals produced multiple absorption peaks with new surface functional groups, proving that the modification of clay minerals with phosphoric acid was successful.

### Clay preparation

PFL-1 (Palygorskite), Gadsden county, Florida, USA; KGa-1b(Kaolinite), Washington County, Georgia, USA; SWy-2(Na-rich Montmorillonite), Crook Country, Wyoming, USA.

### Verifying the validity

**Fig. 1:**
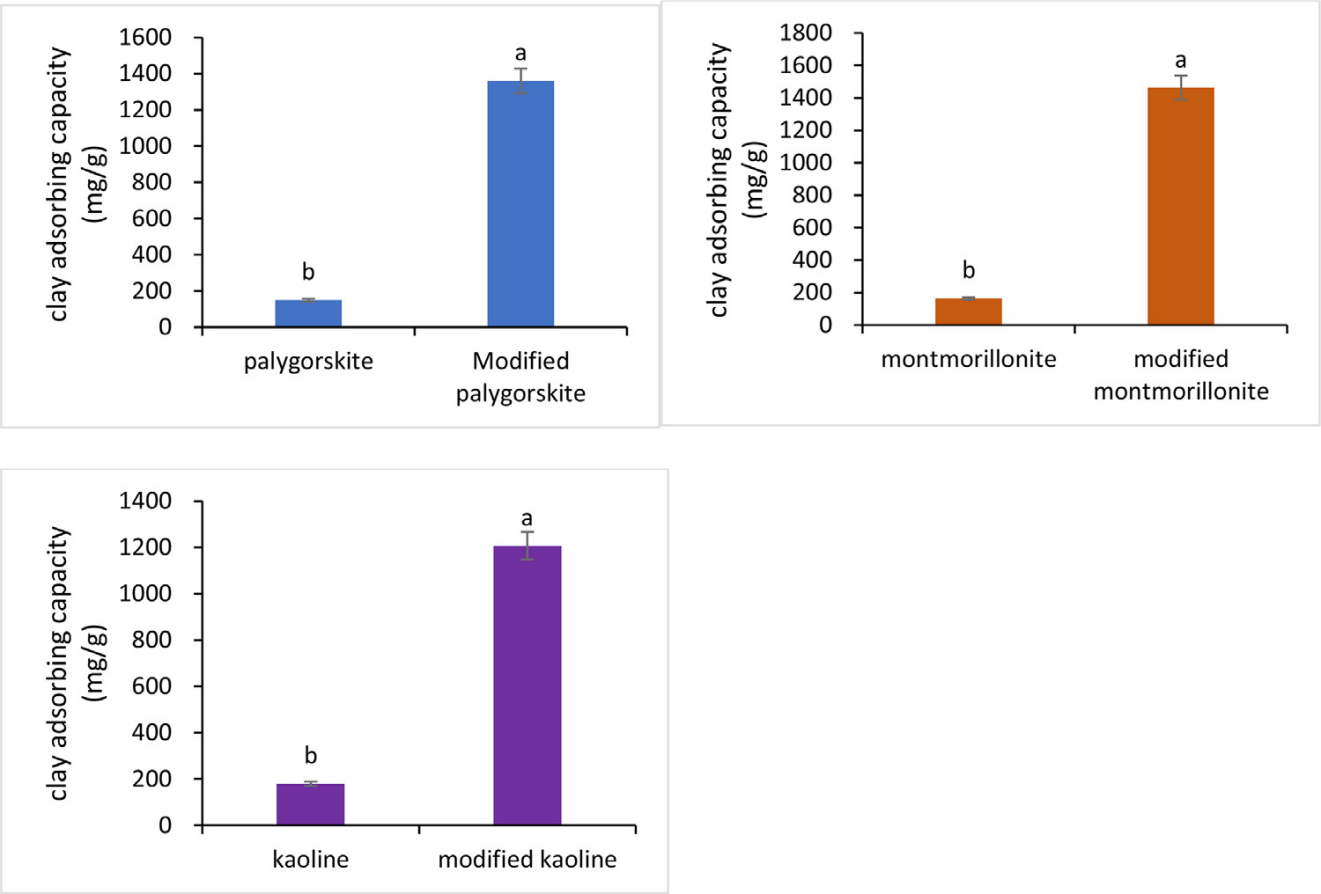
Improvement of uranium adsorption capacity of PO_4_-activated clay minerals.

Fig. 1 shows that phosphoric acid activated clay significantly increased the adsorption capacity of uranium. When the concentration of initial uranium solution was 200 mg/L, the uranium adsorption capacity of phosphoric acid activated palygorskite increased by 9.0 times. Similarly, U adsorption capacity of phosphoric acid modified montmorillonite increased by 8.9 times and that of modified kaolin increased by 6.7 times.

**Fig. 2:**
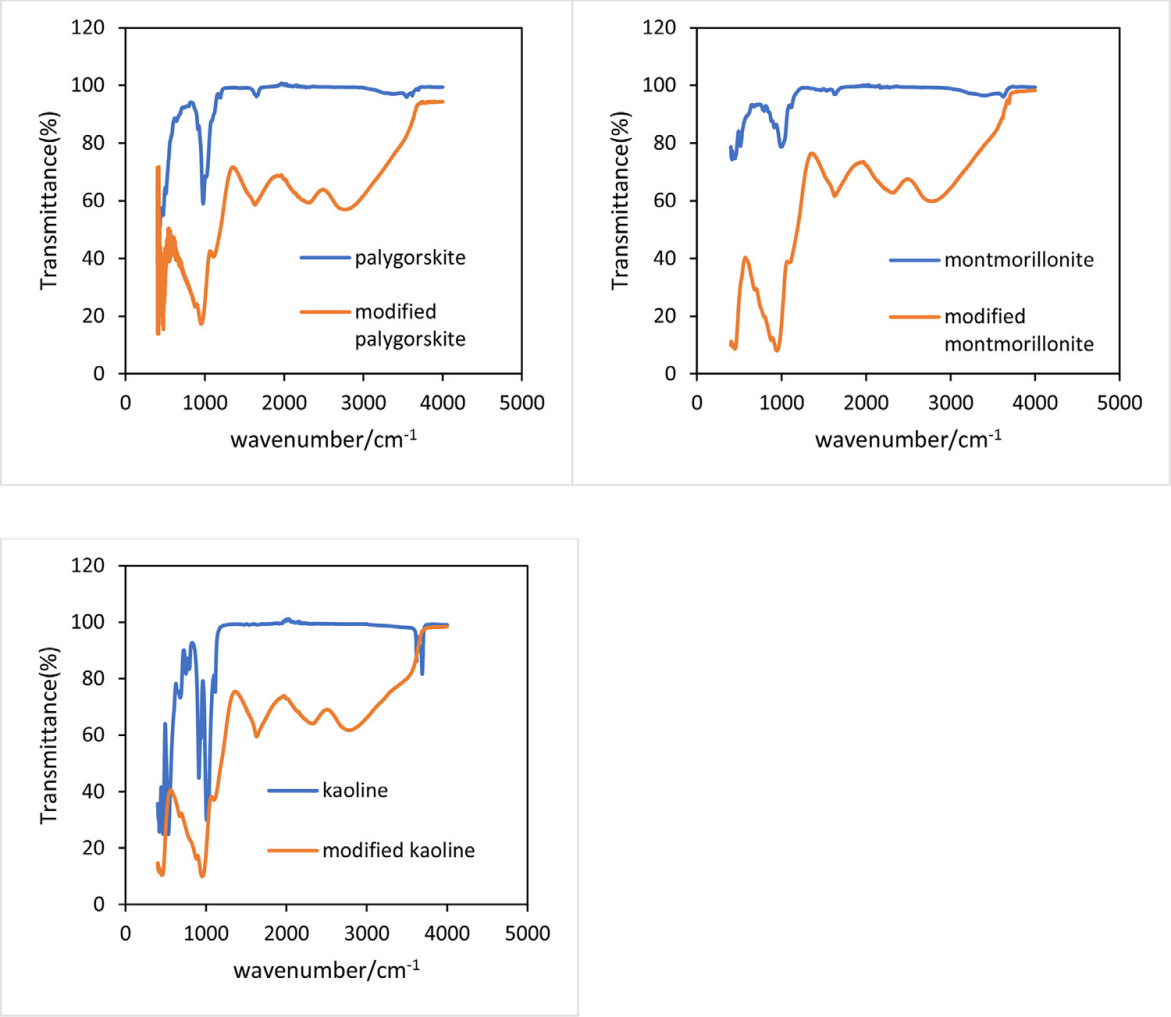
FTIR spectrum of raw and P- activated clay minerals.

Fig. 2 shows that the phosphoric acid-modified clay minerals changed surface functional groups compared to the original minerals. All three clay minerals had an absorption peak of C—O at the wavelength of 1000 cm^−1^, -PO_4_ at 1100 cm^−1^, C—N at 1500 cm^−1^, O=C=O at 2400cm^−1^. -CHO at 2800 cm^−1^, which proved that the modification of clay minerals with phosphoric acid was successful.

## Declaration of Competing Interest

The authors declare that they have no known competing financial interests or personal relationships that could have appeared to influence the work reported in this paper.
